# Supplementary material for: Exploring the Association between Gut Microbiota and Inflammatory Skin Diseases: A Two-Sample Mendelian Randomization Analysis
Source: Microorganisms. 2023 Oct 19;11(10):2586. doi: 10.3390/microorganisms11102586 (PMC10609507; doi:10.3390/microorganisms11102586)
Supplement: Supplementary file 1 [file microorganisms-11-02586-s001.zip › supplementary figures.pdf]

Supplementary Figure S1 Scatter plot analysis of the association between gut microbiota and eczema.

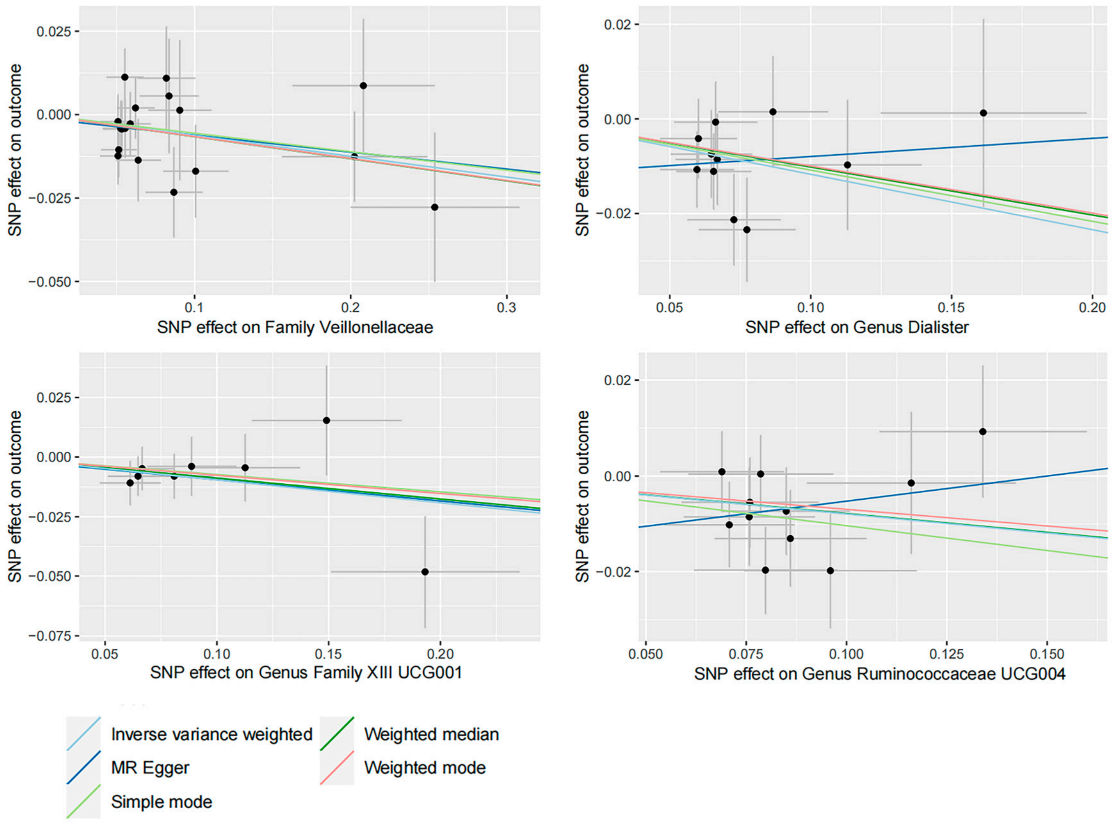

Supplementary Figure S2 Scatter plot analysis of the association between gut microbiota and acne.

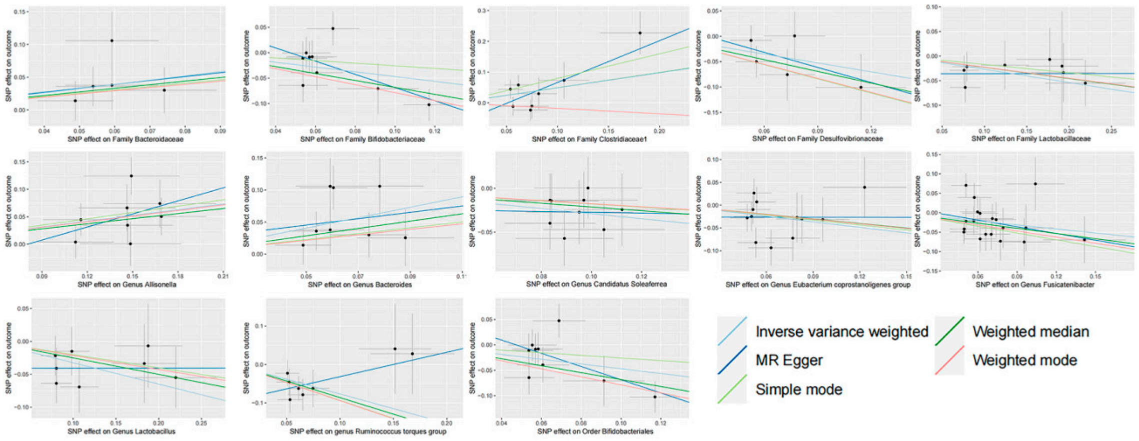

Supplementary Figure S3 Scatter plot analysis of the association between gut microbiota and psoriasis.

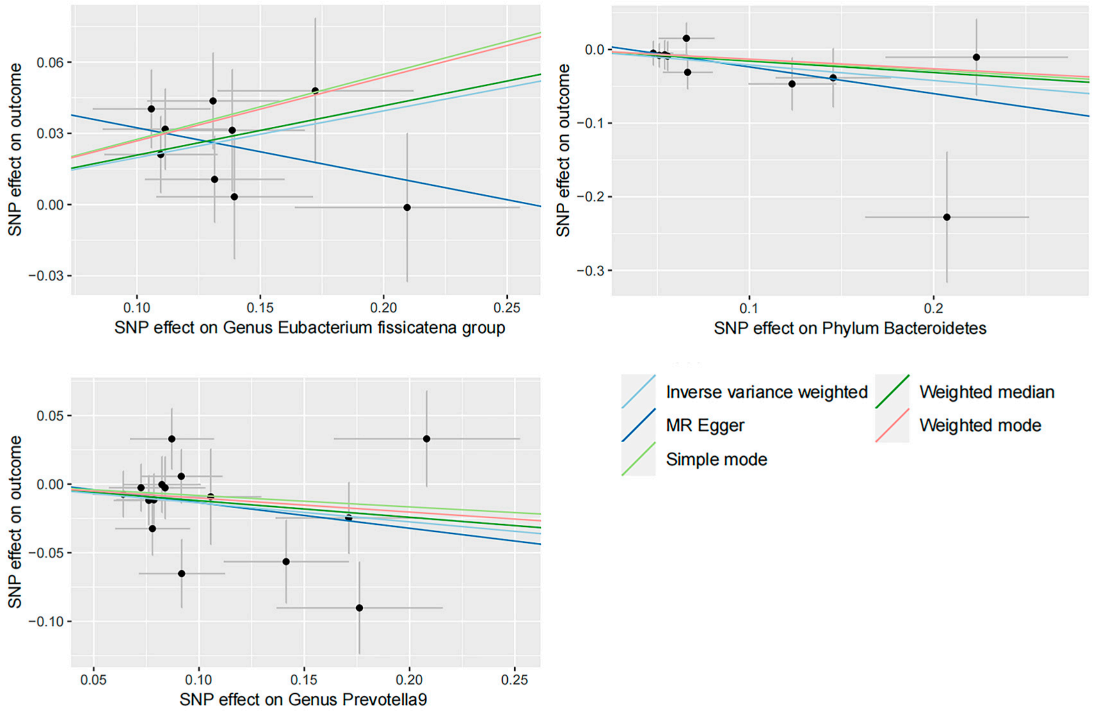

Supplementary Figure S4 Scatter plot analysis of the association between gut microbiota and rosacea.

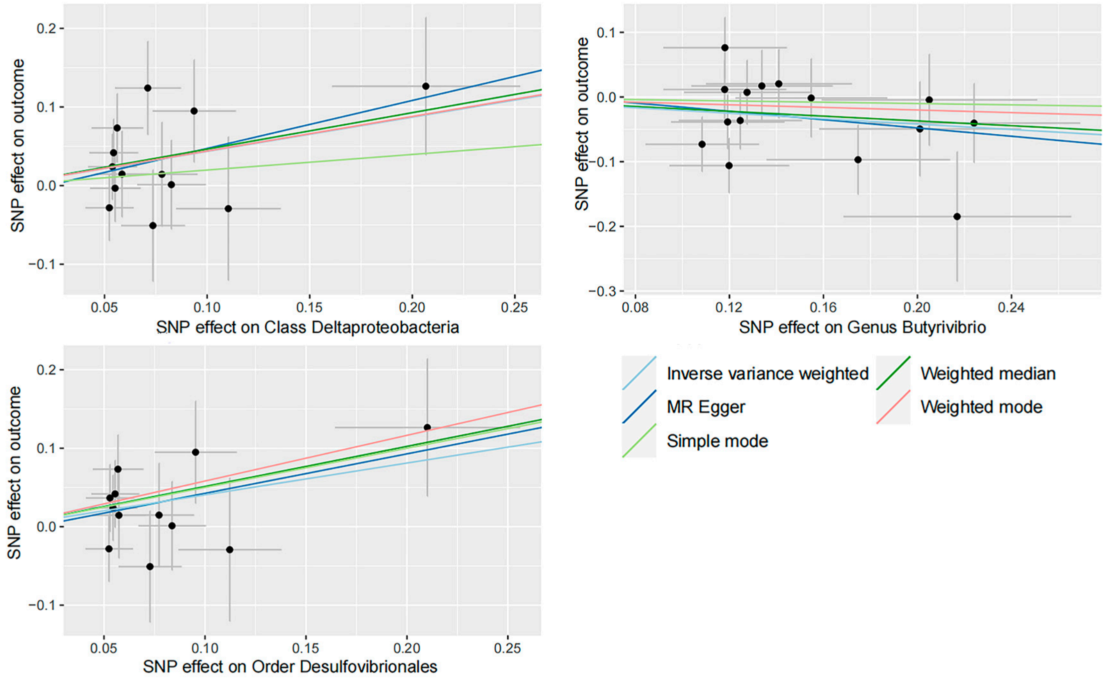

Supplementary Figure S5 Forest plots of SNPs associated with gut microbiota and eczema.

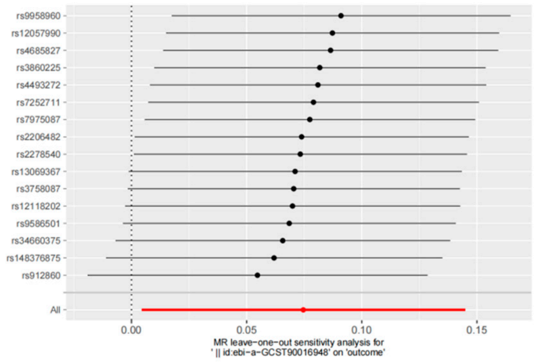

Genus Prevotellaceae

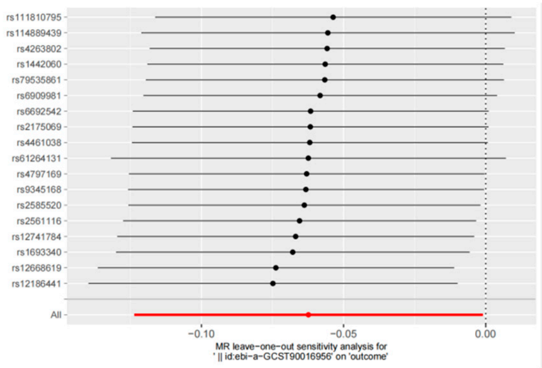

Genus Veillonellaceae

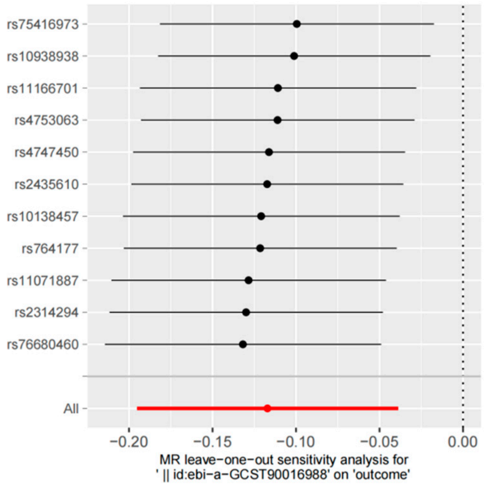

Genus Dialister

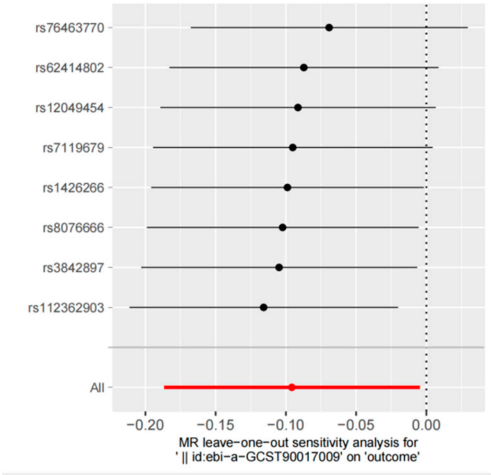

Genus Family XIII

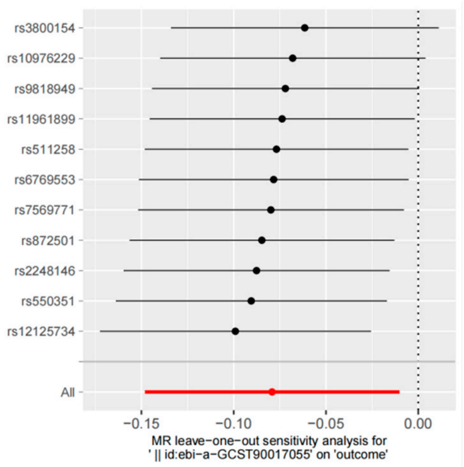

Genus Ruminococcaceae UCG004

Supplementary Figure S6 Forest plots of SNPs associated with gut microbiota and acne.

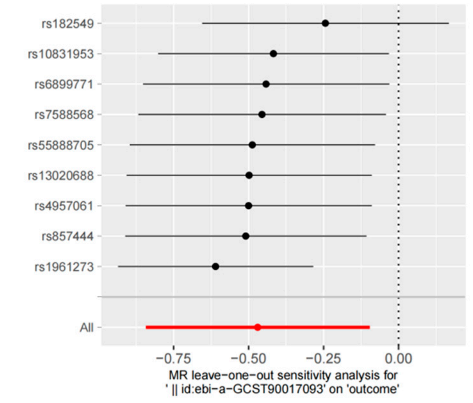

Order Bifidobacteriales

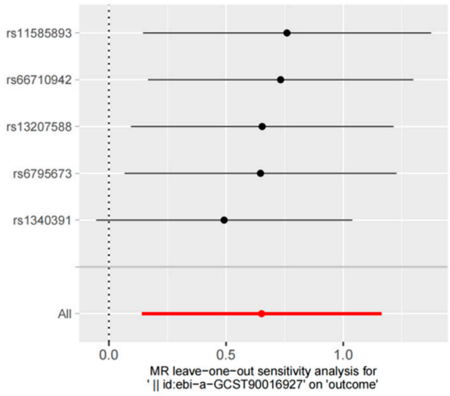

Family Bacteroidaceae

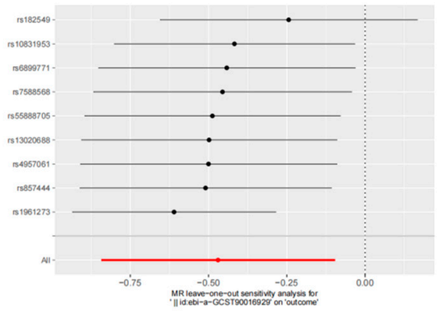

Family Bifidobacteriaceae

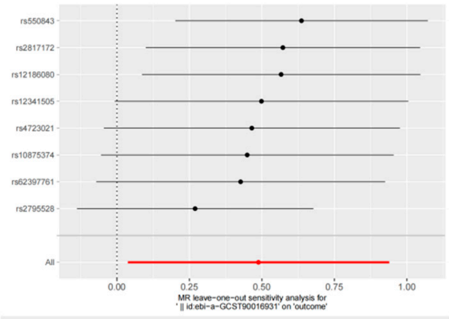

Family Clostridiaceae1

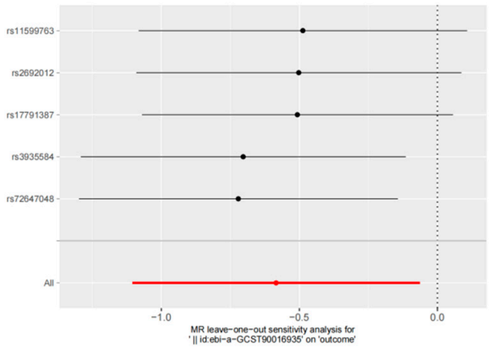

Family Desulfovibrionaceae

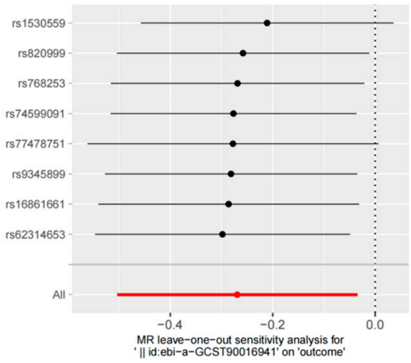

Family Lactobacillaceae

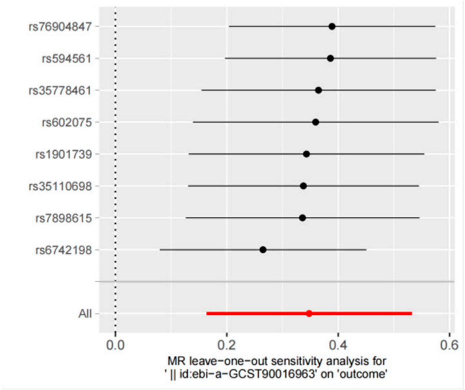

Genus Allisonella

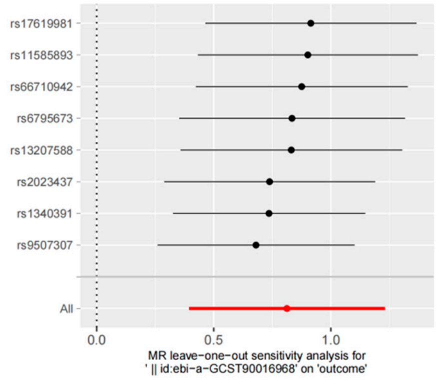

Genus Bacteroides

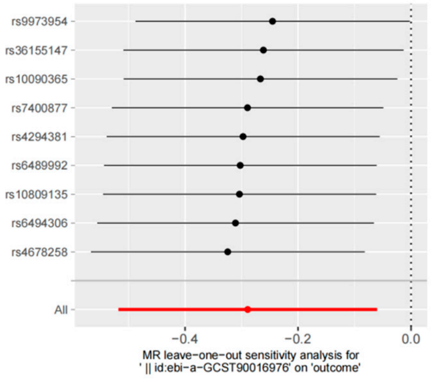

Genus Candidatus Soleaferrea

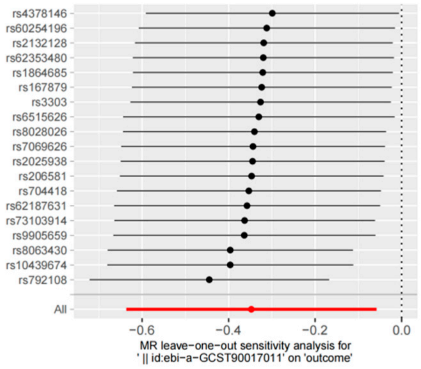

Genus Fusicatenibacter

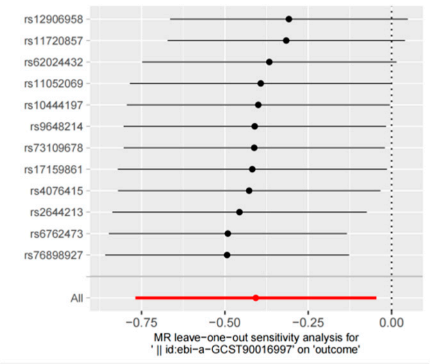

Genus Eubacterium coprostanoligenes group

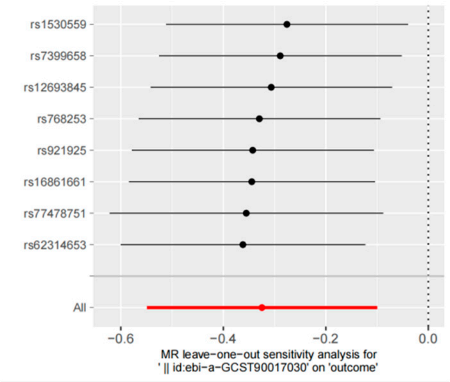

Genus Lactobacillus

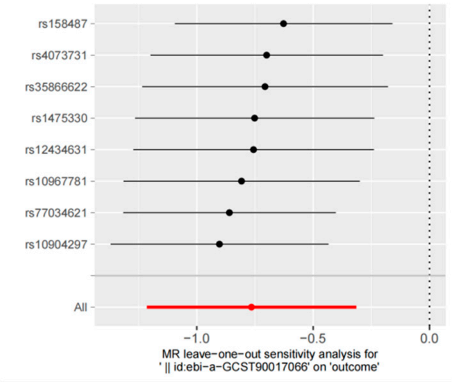

Genus Ruminococcus torques group

Supplementary Figure S7 Forest plots of SNPs associated with gut microbiota and psoriasis.

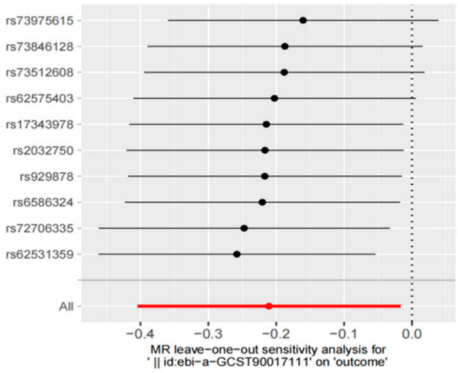

Phylum Bacteroidetes

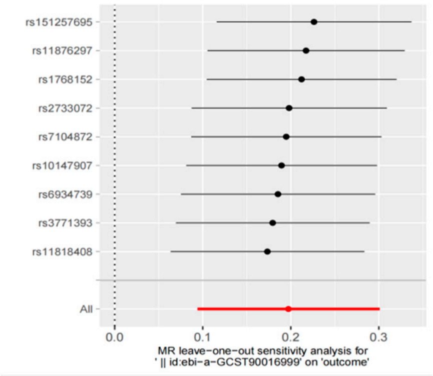

Genus Eubacterium

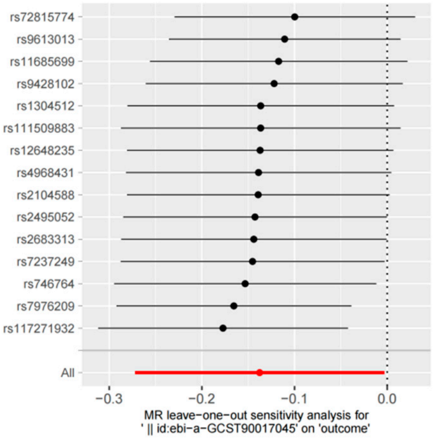

Genus Prevotella9

Supplementary Figure S8 Forest plots of SNPs associated with gut microbiota and rosacea.

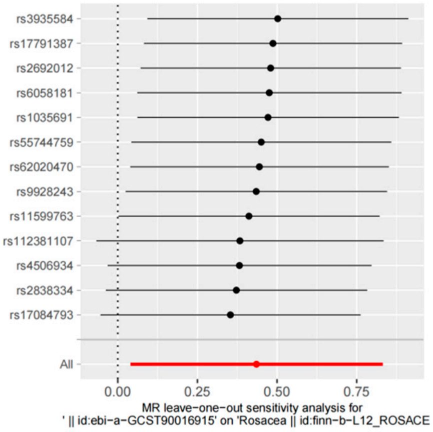

Class Deltaproteobacteria

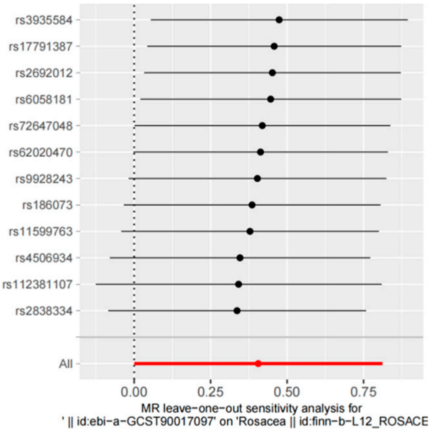

Order Desulfovibrionales

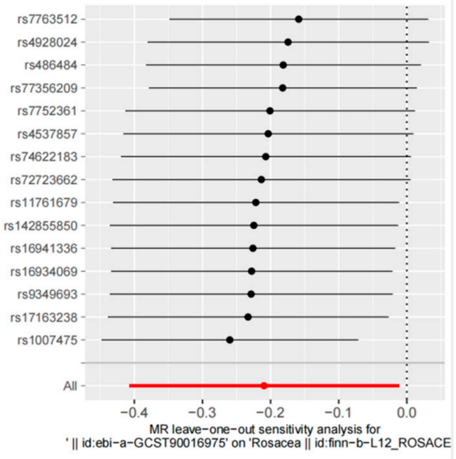

Genus Butyrivibrio
