# Supplementary figures and images for: Exploring the Association between Gut Microbiota and Inflammatory Skin Diseases: A Two-Sample Mendelian Randomization Analysis
Source: Microorganisms. 2023 Oct 19;11(10):2586. doi: 10.3390/microorganisms11102586 (PMC10609507; doi:10.3390/microorganisms11102586)

# MR Method

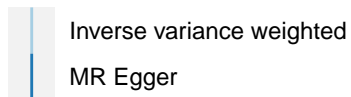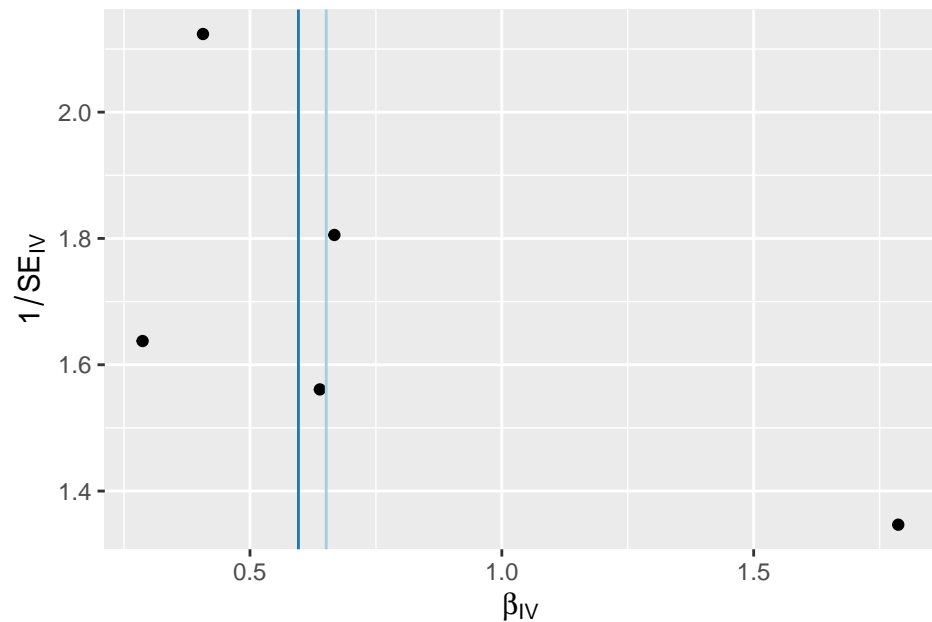

Supplement: Supplementary file 1 [file microorganisms-11-02586-s001.zip › funnel plot/acne_ebi-a-GCST90016927.pdf]

# MR Method

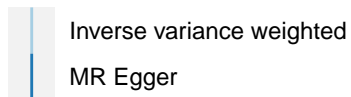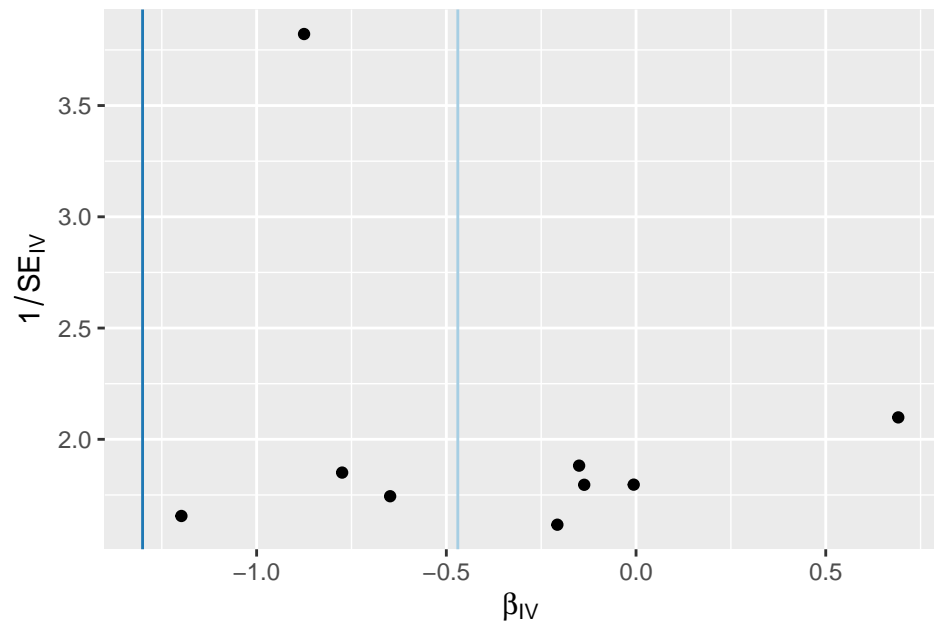

Supplement: Supplementary file 1 [file microorganisms-11-02586-s001.zip › funnel plot/acne_ebi-a-GCST90016929.pdf]

## MR Method

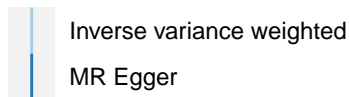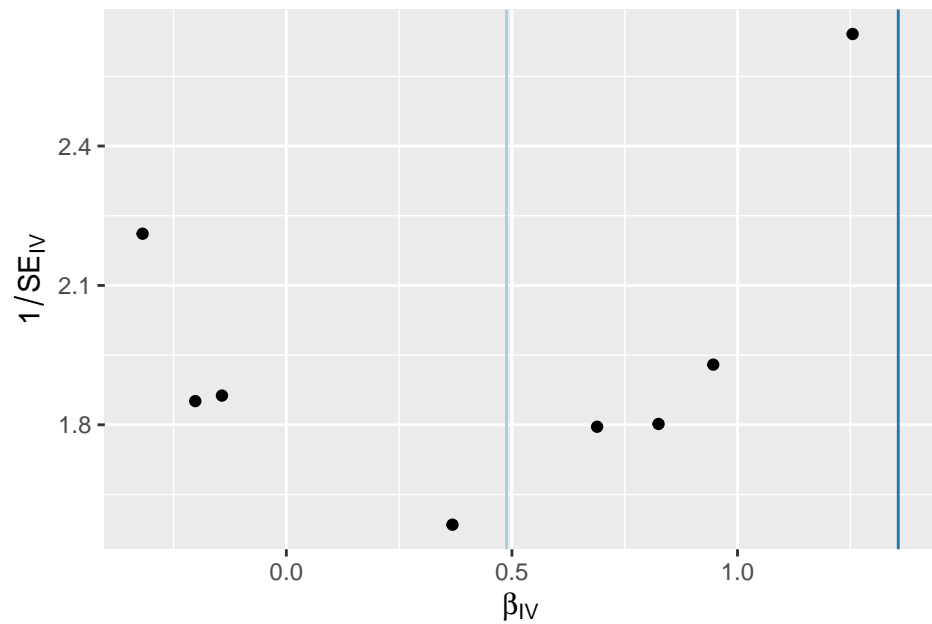

Supplement: Supplementary file 1 [file microorganisms-11-02586-s001.zip › funnel plot/acne_ebi-a-GCST90016931.pdf]

## MR Method

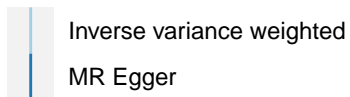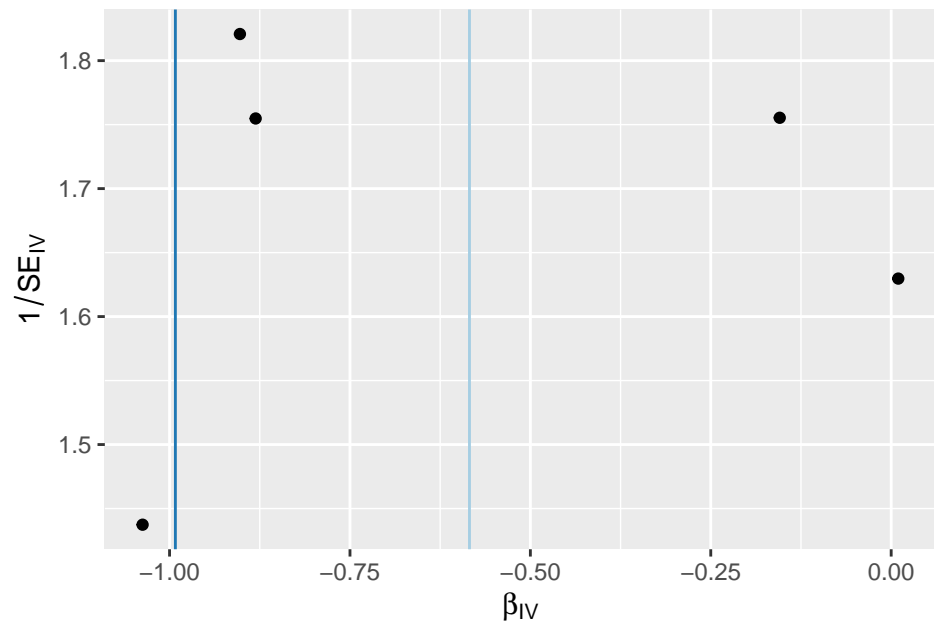

Supplement: Supplementary file 1 [file microorganisms-11-02586-s001.zip › funnel plot/acne_ebi-a-GCST90016935.pdf]

## MR Method

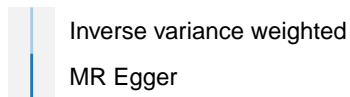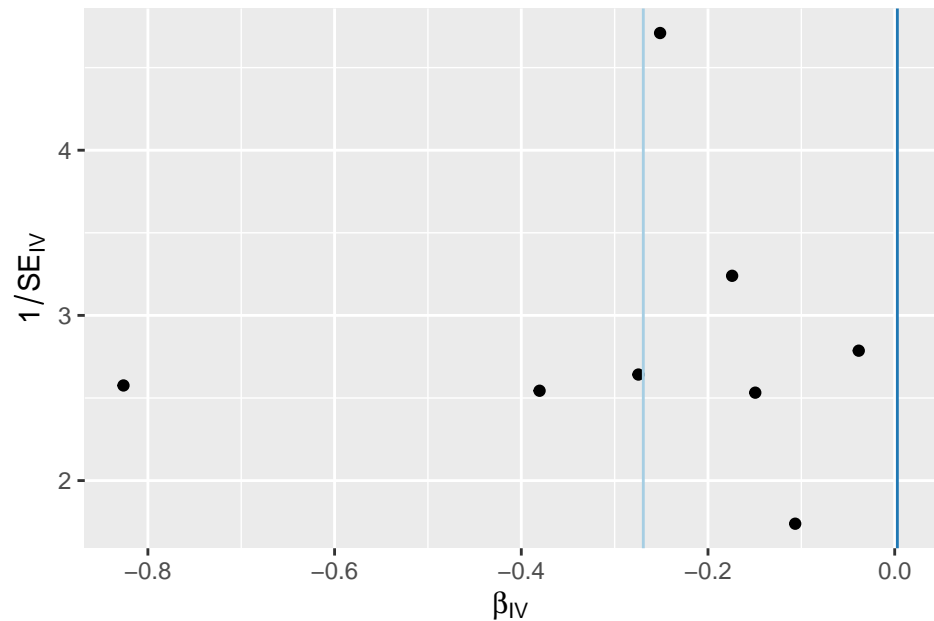

Supplement: Supplementary file 1 [file microorganisms-11-02586-s001.zip › funnel plot/acne_ebi-a-GCST90016941.pdf]

# MR Method

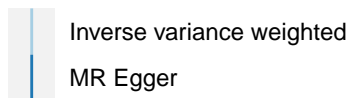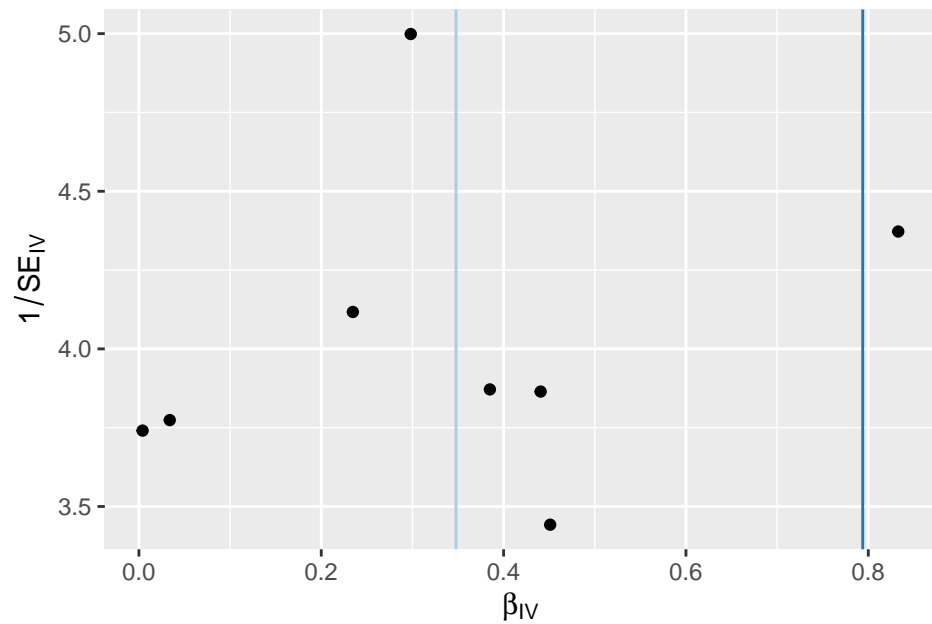

Supplement: Supplementary file 1 [file microorganisms-11-02586-s001.zip › funnel plot/acne_ebi-a-GCST90016963.pdf]

# MR Method

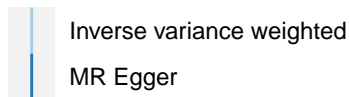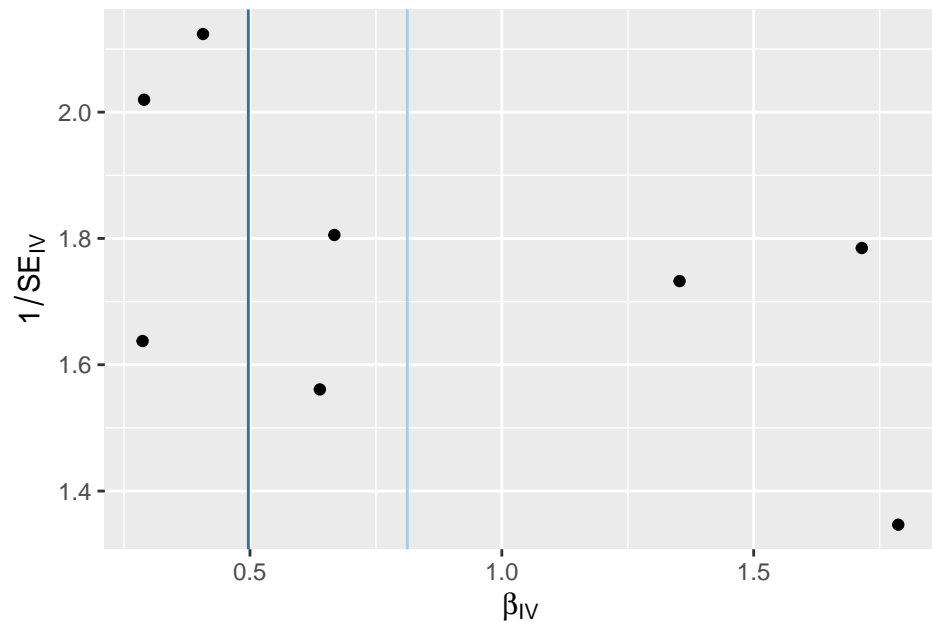

Supplement: Supplementary file 1 [file microorganisms-11-02586-s001.zip › funnel plot/acne_ebi-a-GCST90016968.pdf]

# MR Method

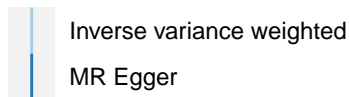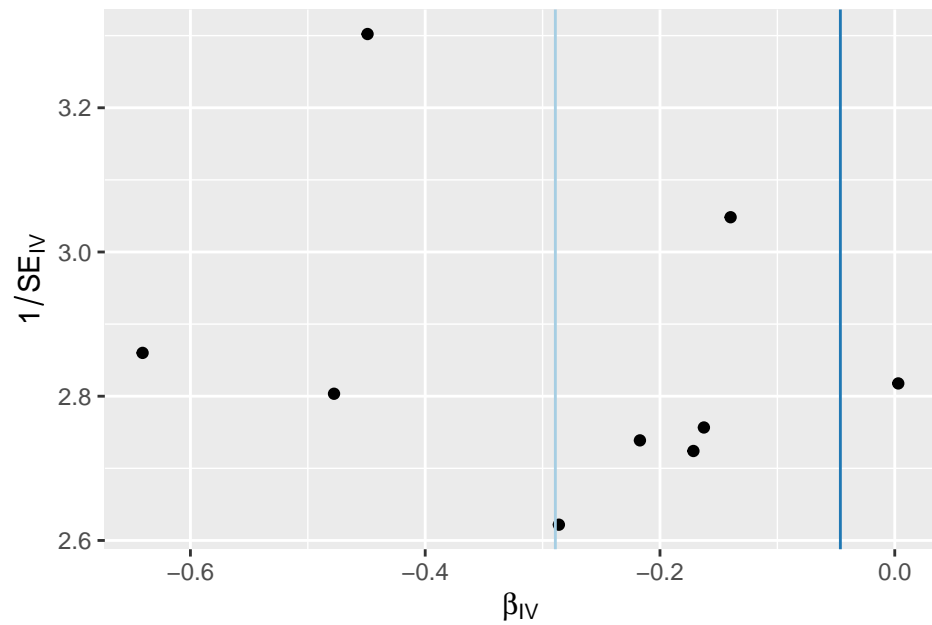

Supplement: Supplementary file 1 [file microorganisms-11-02586-s001.zip › funnel plot/acne_ebi-a-GCST90016976.pdf]

# MR Method

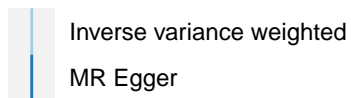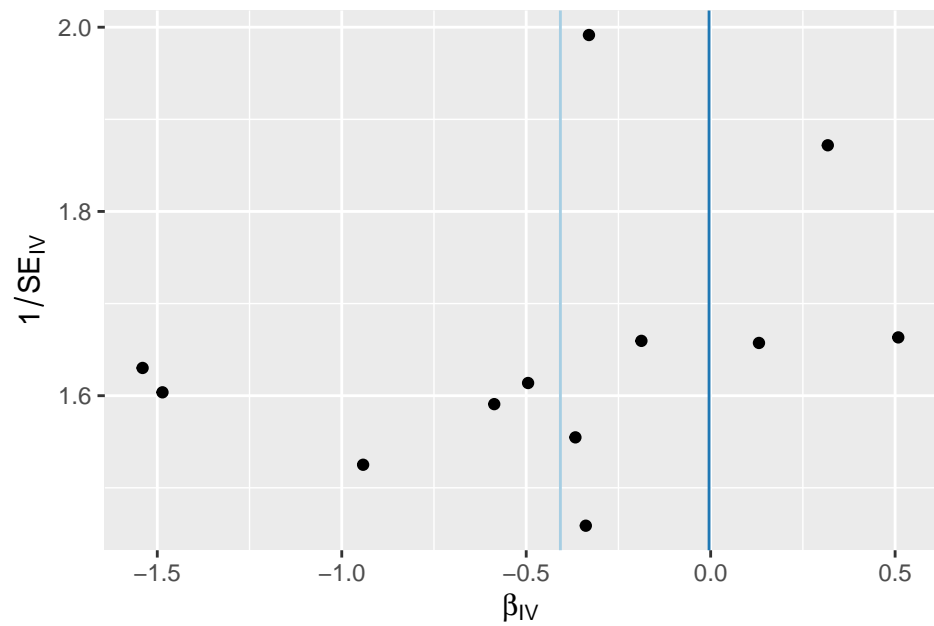

Supplement: Supplementary file 1 [file microorganisms-11-02586-s001.zip › funnel plot/acne_ebi-a-GCST90016997.pdf]

# MR Method

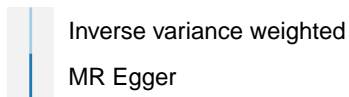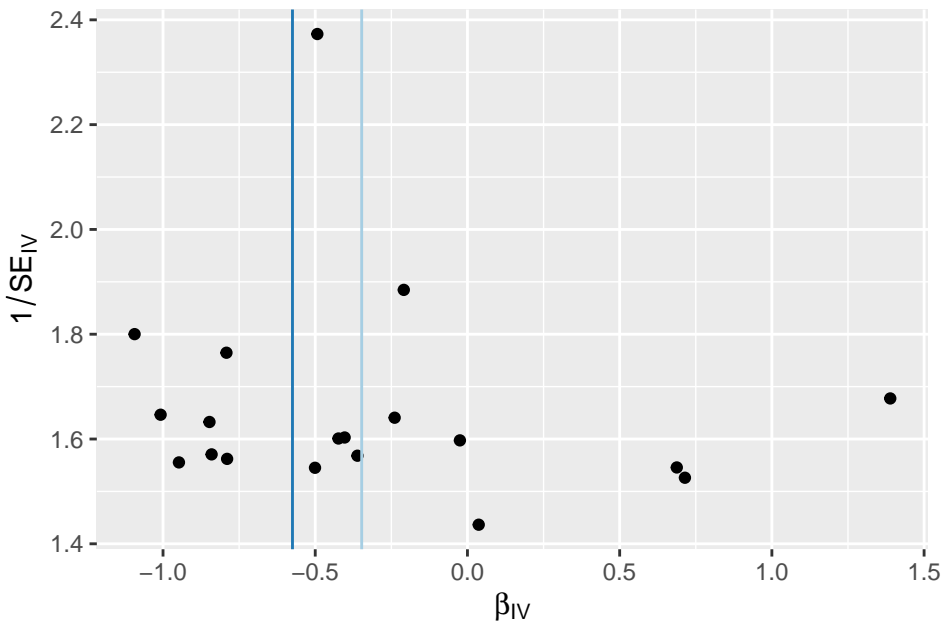

Supplement: Supplementary file 1 [file microorganisms-11-02586-s001.zip › funnel plot/acne_ebi-a-GCST90017011.pdf]

## MR Method

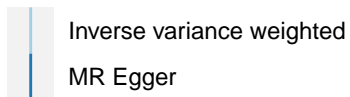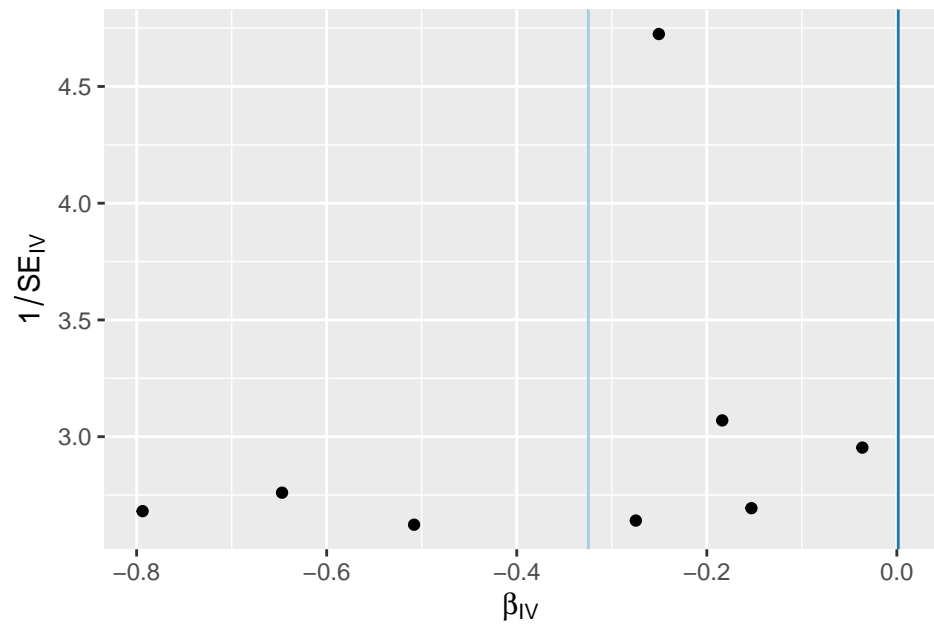

Supplement: Supplementary file 1 [file microorganisms-11-02586-s001.zip › funnel plot/acne_ebi-a-GCST90017030.pdf]

# MR Method

- Inverse variance weighted
- MR Egger

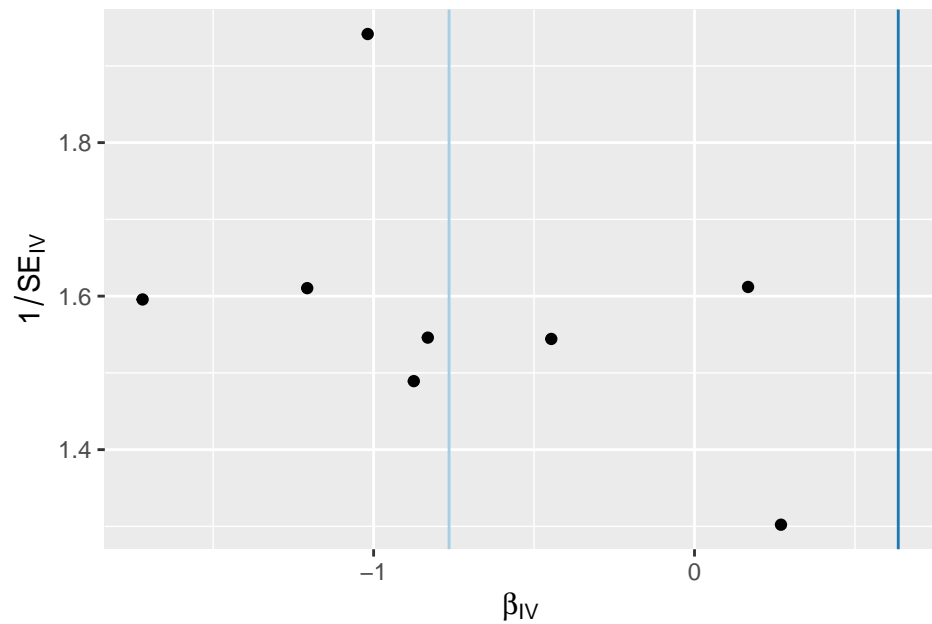

Supplement: Supplementary file 1 [file microorganisms-11-02586-s001.zip › funnel plot/acne_ebi-a-GCST90017066.pdf]

# MR Method

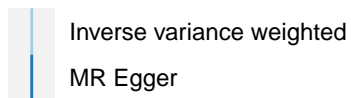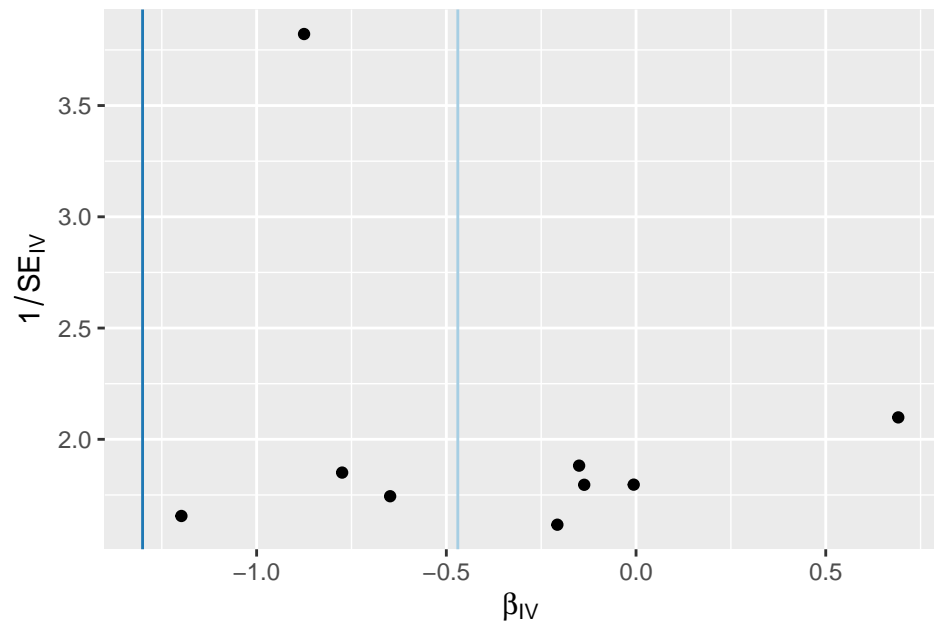

Supplement: Supplementary file 1 [file microorganisms-11-02586-s001.zip › funnel plot/acne_ebi-a-GCST90017093.pdf]

## MR Method

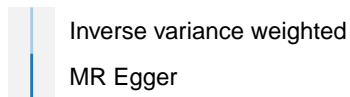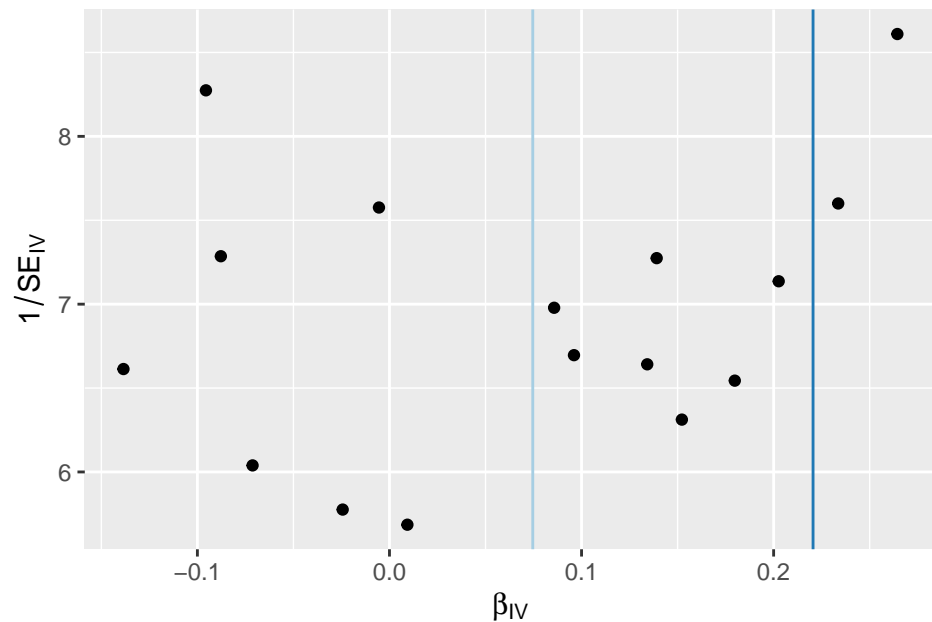

Supplement: Supplementary file 1 [file microorganisms-11-02586-s001.zip › funnel plot/derma_ebi-a-GCST90016948.pdf]

## MR Method

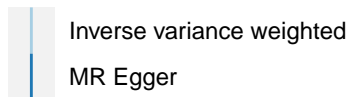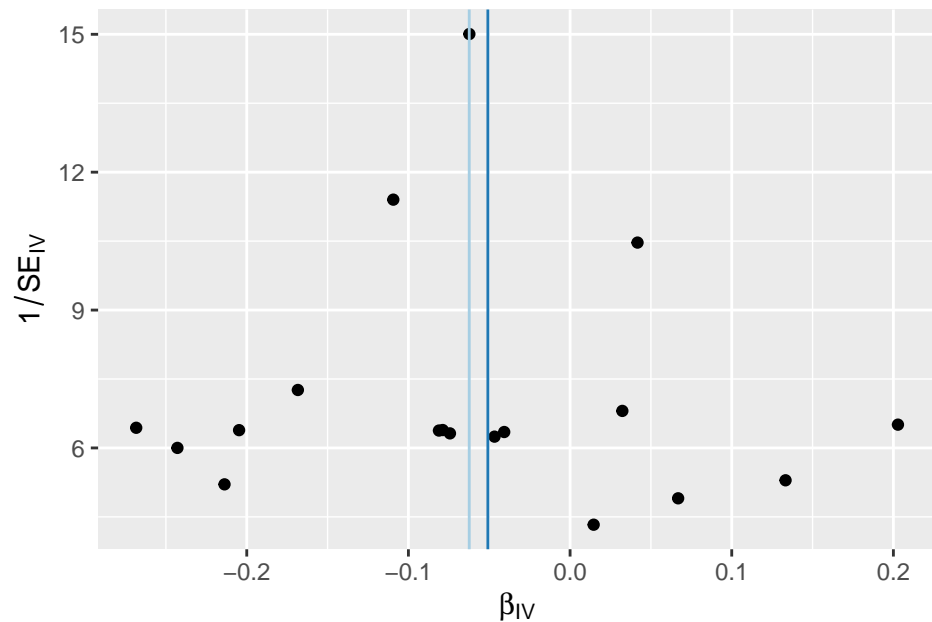

Supplement: Supplementary file 1 [file microorganisms-11-02586-s001.zip › funnel plot/derma_ebi-a-GCST90016956.pdf]

# MR Method

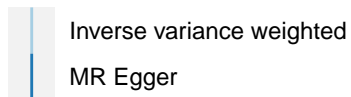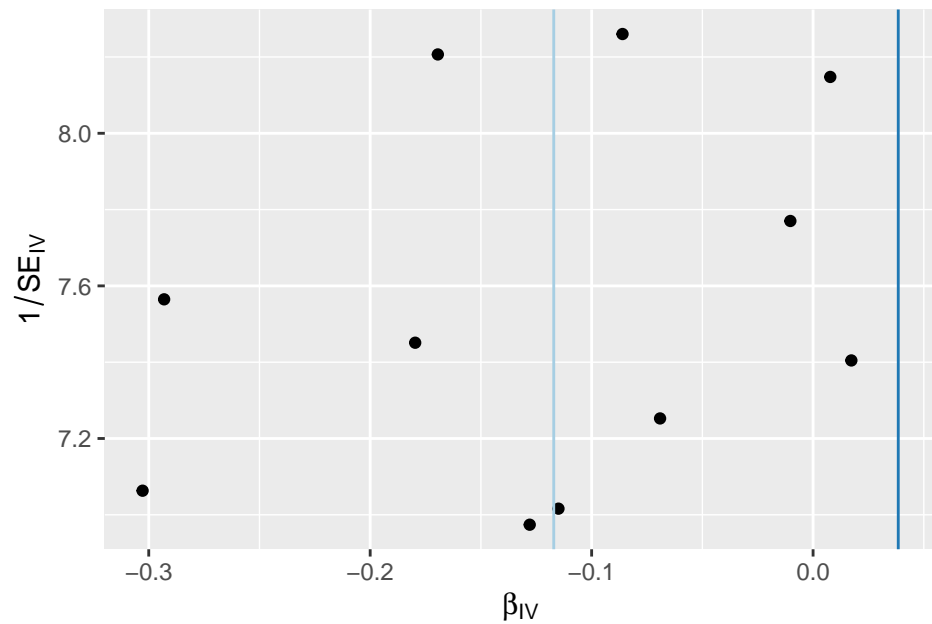

Supplement: Supplementary file 1 [file microorganisms-11-02586-s001.zip › funnel plot/derma_ebi-a-GCST90016988.pdf]

# MR Method

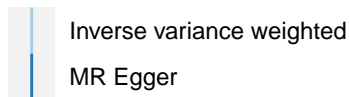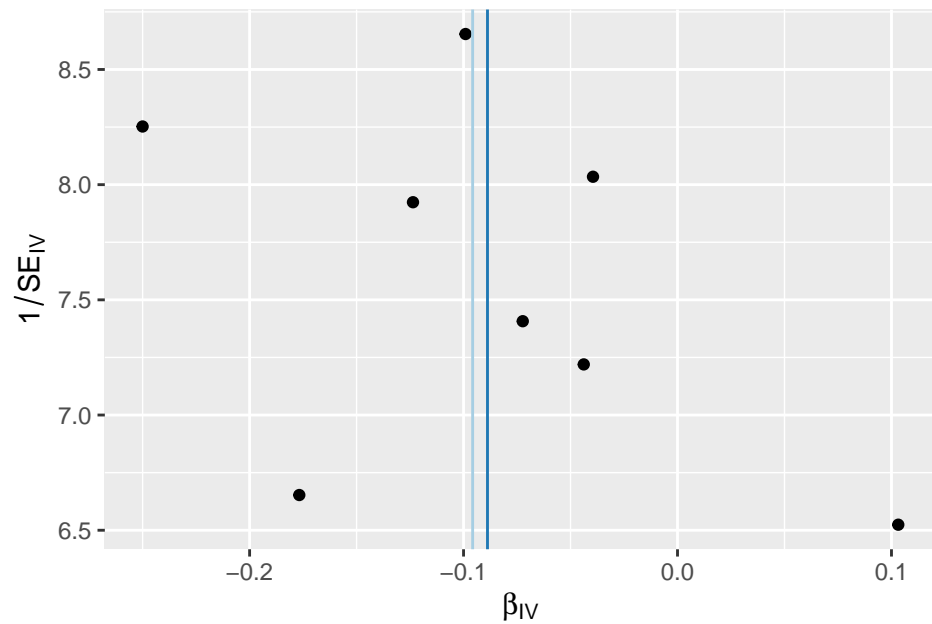

Supplement: Supplementary file 1 [file microorganisms-11-02586-s001.zip › funnel plot/derma_ebi-a-GCST90017009.pdf]

## MR Method

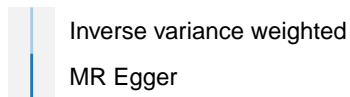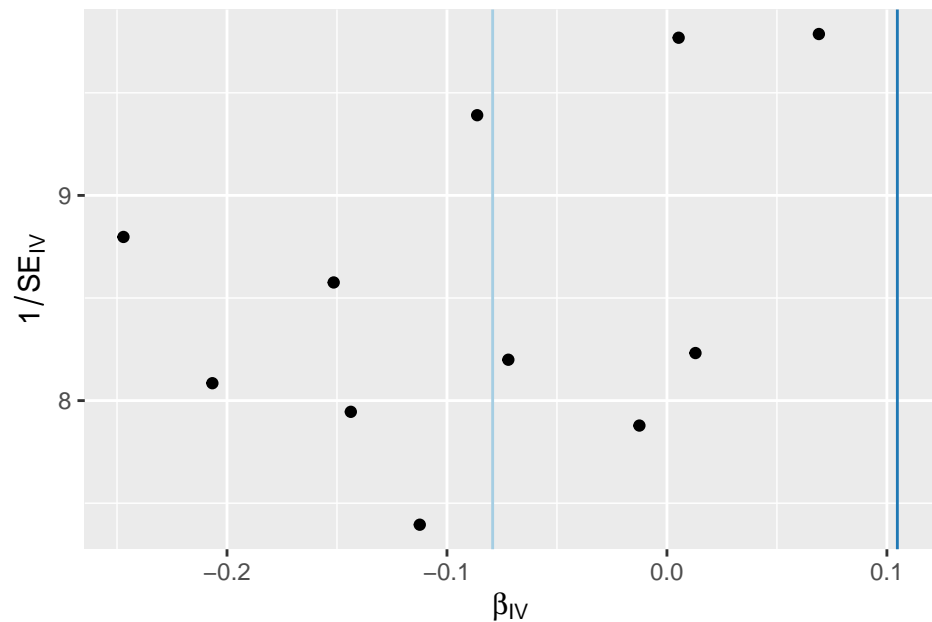

Supplement: Supplementary file 1 [file microorganisms-11-02586-s001.zip › funnel plot/derma_ebi-a-GCST90017055.pdf]

# MR Method

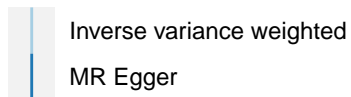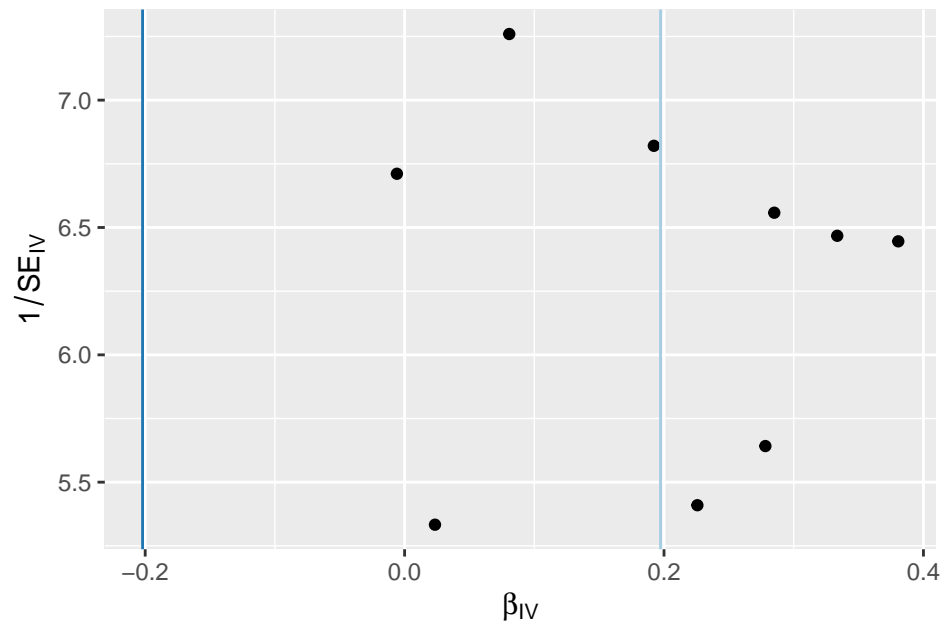

Supplement: Supplementary file 1 [file microorganisms-11-02586-s001.zip › funnel plot/psoriasis_ebi-a-GCST90016999.pdf]

## MR Method

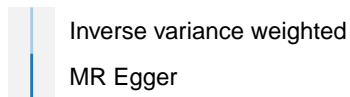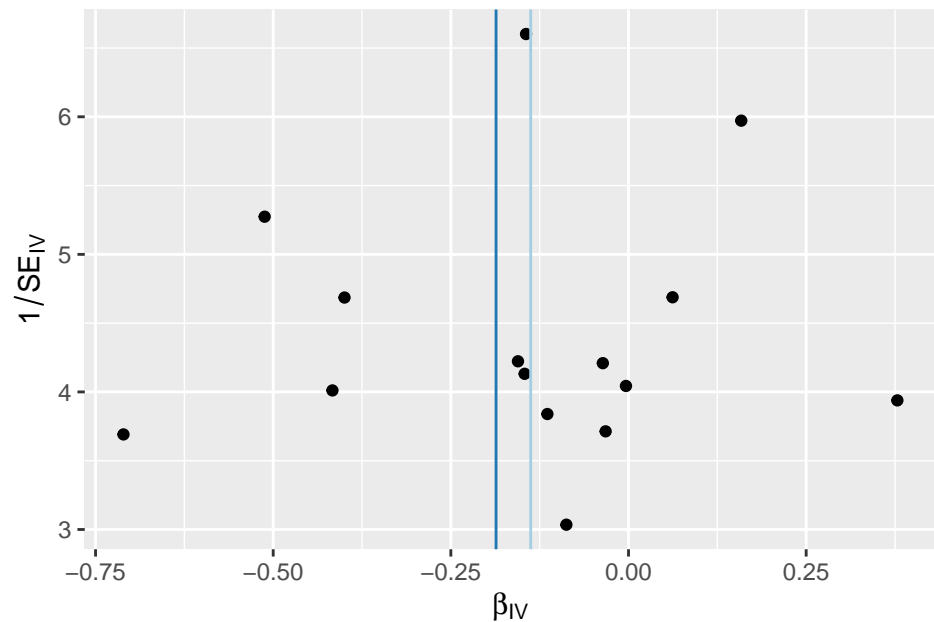

Supplement: Supplementary file 1 [file microorganisms-11-02586-s001.zip › funnel plot/psoriasis_ebi-a-GCST90017045.pdf]

# MR Method

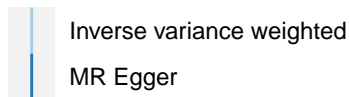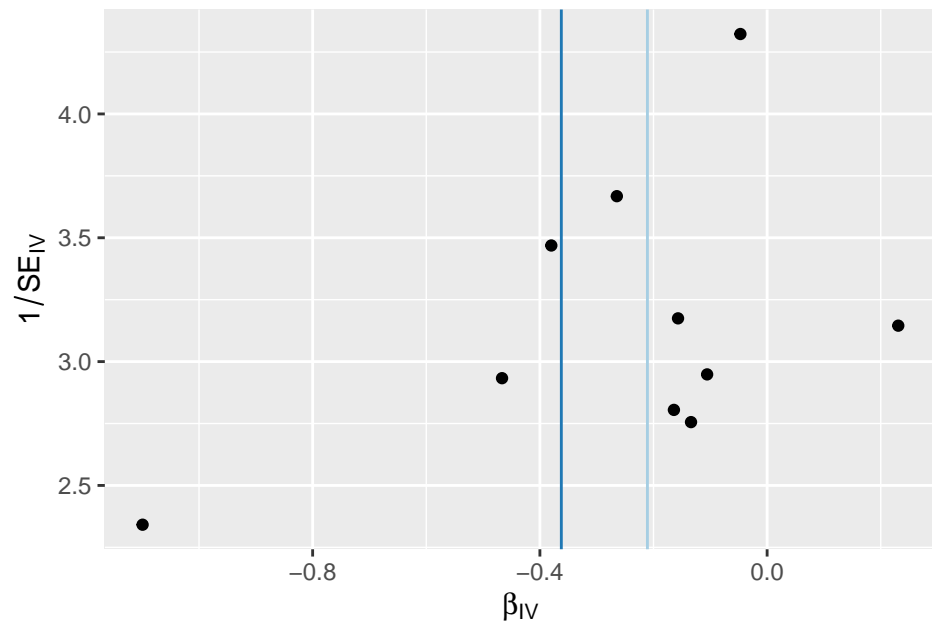

Supplement: Supplementary file 1 [file microorganisms-11-02586-s001.zip › funnel plot/psoriasis_ebi-a-GCST90017111.pdf]

## MR Method

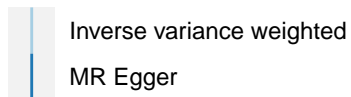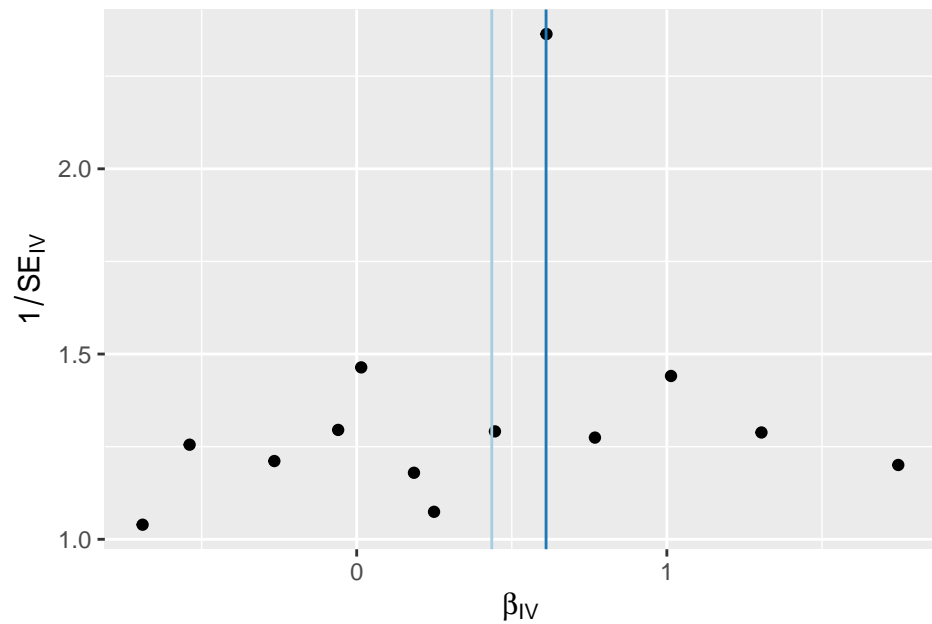

Supplement: Supplementary file 1 [file microorganisms-11-02586-s001.zip › funnel plot/rosacea_ebi-a-GCST90016915.pdf]

# MR Method

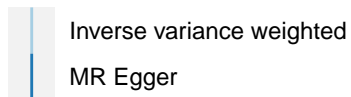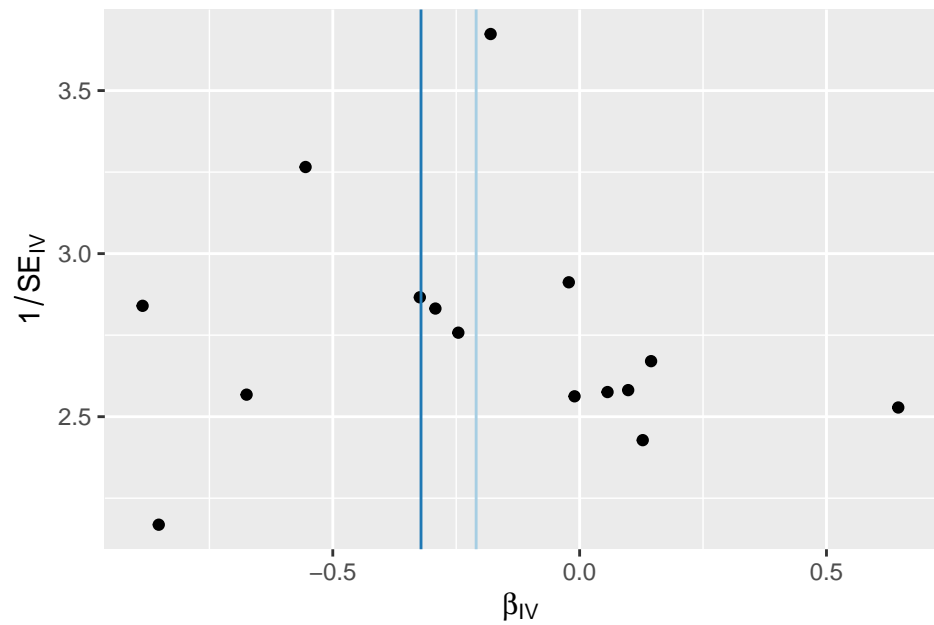

Supplement: Supplementary file 1 [file microorganisms-11-02586-s001.zip › funnel plot/rosacea_ebi-a-GCST90016975.pdf]

# MR Method

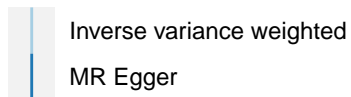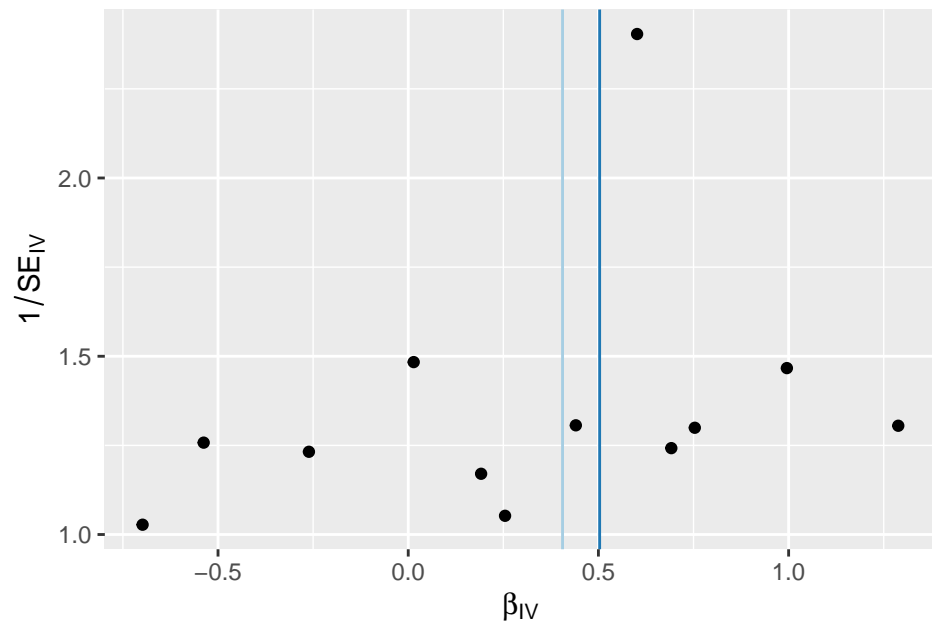

Supplement: Supplementary file 1 [file microorganisms-11-02586-s001.zip › funnel plot/rosacea_ebi-a-GCST90017097.pdf]
